# The Impacts of Acidophilic Lactic Acid Bacteria on Food and Human Health: A Review of the Current Knowledge

**DOI:** 10.3390/foods12152965

**Published:** 2023-08-05

**Authors:** Mehmet Arif Icer, Sena Özbay, Duygu Ağagündüz, Bayram Kelle, Elena Bartkiene, João Miguel F. Rocha, Fatih Ozogul

**Affiliations:** 1 Department of Nutrition and Dietetics, Faculty of Health Sciences, Amasya University, Amasya 05100, Turkey; m.arif.icer@gmail.com; 2 Department of Food Technology, Kaman Vocational School, Kırşehir Ahi Evran University, Kırşehir 40360, Turkey; sena_ozbay@hotmail.com; 3 Department of Nutrition and Dietetics, Faculty of Health Sciences, Gazi University, Emek, Ankara 06490, Turkey; 4 Department of Physical Medicine and Rehabilitation, Faculty of Medicine, Cukurova University, Adana 01330, Turkey; bkelle@cu.edu.tr; 5 Department of Food Safety and Quality, Lithuanian University of Health Sciences Tilzes 18, LT-47181 Kaunas, Lithuania; elena.bartkiene@lsmuni.lt; 6 Institute of Animal Rearing Technologies, Lithuanian University of Health Sciences, Tilzes Street 18, LT-47181 Kaunas, Lithuania; 7 Universidade Católica Portuguesa, CBQF—Centro de Biotecnologia e Química Fina—Laboratório Associado, Escola Superior de Biotecnologia, Rua Diogo Botelho 1327, 4169-005 Porto, Portugal; 8 LEPABE—Laboratory for Process Engineering, Environment, Biotechnology and Energy, Faculty of Engineering, University of Porto, Rua Dr. Roberto Frias, s/n, 4200-465 Porto, Portugal; 9 ALiCE—Associate Laboratory in Chemical Engineering, Faculty of Engineering, University of Porto, Rua Dr. Roberto Frias, s/n, 4200-465 Porto, Portugal; 10 Department of Seafood Processing Technology, Cukurova University, Balcalı, Adana 01330, Turkey; fozogul@cu.edu.tr; 11 Biotechnology Research and Application Center, Cukurova University, Adana 01330, Turkey

**Keywords:** lactic acid bacteria, probiotics, beneficial compounds, toxic compounds, human health, safety assessment

## Abstract

The need to improve the safety/quality of food and the health of the hosts has resulted in increasing worldwide interest in acidophilic lactic acid bacteria (LAB) for the food, livestock as well as health industries. In addition to the use of acidophilic LAB with probiotic potential for food fermentation and preservation, their application in the natural disposal of acidic wastes polluting the environment is also being investigated. Considering this new benefit that has been assigned to probiotic microorganisms in recent years, the acceleration in efforts to identify new, efficient, promising probiotic acidophilic LAB is not surprising. One of these effots is to determine both the beneficial and harmful compounds synthesized by acidophilic LAB. Moreover, microorganisms are of concern due to their possible hemolytic, DNase, gelatinase and mucinolytic activities, and the presence of virulence/antibiotic genes. Hence, it is argued that acidophilic LAB should be evaluated for these parameters before their use in the health/food/livestock industry. However, this issue has not yet been fully discussed in the literature. Thus, this review pays attention to the less-known aspects of acidophilic LAB and the compounds they release, clarifying critical unanswered questions, and discussing their health benefits and safety.

## 1. Introduction

The use of living microorganisms for beneficial purposes on the host dates back to ancient times [1]. Among these microorganisms, those that adapt to living in an acidic pH are called low-pH microorganisms [2]. Acidophilic lactic acid bacteria (LAB) are the most commonly used low-pH microorganisms in the healthcare, pharmaceutical, and food industries [3].

As a new benefit of probiotic microorganisms for human health and the food industry has been presented in recent years, the acceleration in the efforts to identify new, efficient, promising probiotic acidophilic LAB is not surprising [4,5,6].

Fermentation, which increases the shelf life and microbiological safety of foods, as well as making some foods more digestible, is a widely applied method [7]. At present, fermentation is completed under controlled conditions with carefully selected strains [8]. Acidophilic microorganisms are generally used in the production of fermented products [9]. In this respect, the identification of new acidophilic–aciduric microorganisms, which are effective and can be safely applied in the fermentation process, can improve food product quality.

The beneficial effects of some acidophilic LAB and traditional fermented foods on human health are mostly attributed to the compounds they release, such as organic acids, some B-group vitamins, gamma-aminobutyric acid (GABA), amylase enzyme, and bacteriocins [10,11,12]. However, the presence of some toxic compounds (such as biogenic amines) released by acidophilic LAB is also known [13,14]. The identification of acidophilic strains that release beneficial compounds and do not release harmful compounds can contribute to the uses of acidophilic LAB, especially in the health and food industry. Thus, it is thought that acidophilic microorganisms, whose beneficial effects and safety have been determined, may be a new strategy for the bio-enrichment of foods and a cost-effective alternative to existing fortification programs.

New application areas of acidophilic LAB are rapidly being discovered and developed. For example, the natural disposal of acidic wastes polluting the environment as a result of many human activities, especially industrial processes, by acidophilic microorganisms is one of the innovative solutions being developed [15,16].

The safety of all microorganisms, including acidophilic LAB, should be evaluated and confirmed before use [5,17]. Non-hemolytic, non-DNase, non-gelatinase, non-mucinolytic activities, and the absence of virulence/antibiotic-resistance genes, are considered safety prerequisites for the selection of probiotic strains [18,19,20,21,22]. The transfer of unwanted genes to pathogenic bacteria is one of the crucial health risks that are emphasized [18,19,20,21]. As the possible health risks of acidophilic LAB are being determined, it is becoming increasingly important to evaluate their safety and reveal safe strains before their use in the food industry, livestock, and health sector.

Although there are some studies examining the usage areas, health benefits, released compounds, and safety concerns of acidophilic LAB in the literature, the absence of a comprehensive review, in which they are evaluated together, reveals the importance of this review. Therefore, the current review focuses on the unknown aspects of acidophilic LAB, clarifies critical unanswered questions, and pioneers the development of new alternative strategies/functional products based on acidophilic probiotic LAB and their metabolites.

## 2. Overview of Acidophilic Microorganisms

Microorganisms are a group of living creatures that develop or adapt to very different environmental conditions. The conditions under which these creatures optimally develop relate to acidity, high/low temperature, salt concentration, etc. [23]. Some of the microorganisms prefer to live in an acidic environment (acidophilic organisms), while others prefer an alkaline pH (alkaliphilic organisms) [24]. Acidophiles living optimally below the pH-neutral level (7.0) are also divided into different subgroups. Among these, acid-tolerant ones can live above pH 5.0, while optimum conditions are pH 3.0 or below for extreme acidophiles. However, moderate acidophiles reach optimum living conditions between pH 3.0 and 5.0, and hyper-acidophiles reach optimum living conditions below pH 1.0 [2].

Extremophiles are commonly defined as microorganisms that can survive under conditions of severe heat, pH, salt concentration, etc., including extreme acidophiles. These organisms are a significant subject for an innovative study. The idea is inspired by the potential to use these microorganisms in biotechnological and industrial applications, which increases interest in this topic. Very acidic wastes are released into the environment as a result of many human activities, particularly mining, construction, and other industrial processes. The numerous hazardous compounds in these acidic wastes and wastewater are harmful to both people and other organisms. Acidophilic microorganisms are considered good options for the storage of these wastes [15].

Only two species of extreme acidophilic microorganisms had been isolated and characterized by the middle of the 20th century, while, by the beginning of the 21st, more than 50 species had been identified. As they can use different energy sources (solar, organic, and inorganic chemicals), electron-acceptors (oxygen, ferric iron, and sulfur), and carbon sources, acidophiles as a group are now known to be extremely physiologically and metabolically heterogeneous [2].

The main reason why low, acidic conditions are optimal for acidophiles is their cellular adaptation to regulate pH. Many extracellular enzymes derived from acidophiles are known to be functional at a much lower pH than cytoplasmic pH. It is also stated that enzymes such as amylases, proteases, ligases, cellulases, xylanases, α-glucosidases, endoglucanases and esterases, obtained from acidophilic microorganisms, are stable at a low pH [25]. Although they can only grow at pH 2–4, acidophilic bacteria can maintain their cytoplasmic pH at or above 6.0. It is essential to understand the mechanism by which this significant pH difference (ΔpH) is maintained. It is well-known that maintaining this significant transmembrane pH demands energy and that the transmembrane electric potential has to be different from that of neutrophilic bacteria [26]. The highly impermeable cell membrane is one of the many mechanisms that acidophilic microorganisms use to restrict the passage of protons in order to survive in acidic conditions [27]. For instance, both *Thiobacillus acidophilus* and *Bacillus acidocaldarius* showed a reverse transmembrane potential. The cytoplasmic pH can remain constant thanks to this transmembrane potential. *L. acidophilus* has a high cytoplasmic buffering capacity and is responsible for pH homeostasis. When a large influx of H^+^ occurs, the cytoplasmic buffering capacity prevents drastic changes in pH. The resulting increase in positive membrane potential due to this H+ influx eventually leads to the cessation of further H^+^ fluxes [26,28].

There are numerous species and subspecies of acidophilic bacteria, as well as many acidophilic microorganisms, surviving under extremely acidic conditions. The pH levels in the food matrix, however, are not as high as those found in harsh natural environments. For this reason, LAB and their subspecies are of great importance as acidophilic microorganisms in the food matrix. Important acidophilic species can mainly be found in the *Lactobacillus* subgroup.

LAB are rod, cocci and coccobacillus, Gr (+), immobile, catalase (−), microaerophilic or anaerobic microorganisms, with many strains and species. They are also acid-resistant, strongly fermentative, do not reduce nitrates, and need glucose and ammonium as well as some vitamins and amino acids for their growth and development [3,29] Lactobacilli are also a group of Gram-positive, acid-tolerant, non-sporulating, non-respiratory, rod or cocci-shaped bacteria that share common metabolic and physiological characteristics with LAB. These bacteria are commonly found in rotting plants and dairy products, producing lactic acid as the main metabolic product of carbohydrate fermentation [9].

For food fermentation and preservation, LAB are the most frequently used microorganisms. While consuming food, they undergo basically safe metabolic processes that use the available carbohydrates to produce organic acids and other metabolites. They are crucial for the food industry in this regard [3].

LAB, which are frequently used as a starter in foods, follow three main metabolic pathways during fermentation. These are glycolysis (sugar fermentation), lipolysis (degradation of fats) and proteolysis (degradation of proteins). The development of the aroma of dairy products, especially as a result of proteolytic activity, is also important for the food industry [30]. Studies have shown that microorganisms can improve and increase the production of vitamins and flavors, as well as the acidification rate and acid tolerance [31]. Organisms adapted to endure extreme pH conditions have also been shown to be suitable for industrial applications. Many acidophilic microorganisms, some adapted to life at high temperatures, naturally produce enzymes that can degrade polymeric or oligomeric carbon sources [16]. These properties make them preferable for applications in lignocellulosic biorefineries, as well as in the food and textile industries [25].

The proper classification and monitoring of acidophilic metabolic processes also prevents the generation of undesirable metabolic byproducts during food production. For instance, it is vital to know how to control fermentation to prevent the formation of lactic acid and undesirable byproducts. It is also essential to know how LAB work in drinks such as wine and acidic fruit juices [32].

## 3. Acidophilic Lactic Acid Bacteria in Foods

### 3.1. Their Utilization in Fermentation Technology as Starter Cultures

The preservation of foods by fermentation is an ancient, widely practiced method. Fermentation increases the shelf life and microbiological safety of a food [33]. Fermentation also yields new products.

The production of these foods in the past was traditional and secretive due to the long history of fermented foods. Today, fermentation is carried out under controlled conditions with strains that have been carefully chosen [32]. Traditionally, fermentation was carried out by inoculation from the previous batch. However, at present, there are starter cultures for fermentation, thus standardizing the process and product quality.

LAB play a role in the fermentation processes of milk, meat, grains and vegetables owing to their metabolic properties [33]. However, the optimum pH for the growth of most LAB is close to neutral. Therefore, it should be noted that most LAB are neutrophilic. However, some bacterial species, such as Lactobacillus and Oenococcus, show more acidophilic behavior [34]. *Lactobacilli* are usually aciduric or acidophilic. These microorganisms are also strictly fermentative, oxygen-tolerant or anaerobic and have complex nutritional requirements, including carbohydrates, amino acids, peptides, fatty acid esters, salts, nucleic acid derivatives and vitamins [35].

LAB are also significant in animal foods. In the past, straw was used to store grass for use as animal feed. At present, animal feed preserves more nutrients when converted into silage. This process was only made possible when the fermentation of lactic acid bacteria was understood [32]. In this way, acidophilic LAB are used as a starter culture in the production of food. 

*Lactobacillus delbrueckii* subsp. *Bulgaricus,* used as a starter culture, is an important Lactobacilli. This microorganism is a Gram-positive rod, non-motile, and does not form spores. It is considered either aciduric or acidophilic. It requires a low pH (approximately 5.4–4.6) to grow effectively in the optimum temperature range from 43 to 46 °C [9]. *Lactobacillus delbrueckii* subsp. *bulgaricus* is widely used together with *Streptococcus thermophilus* as a starter culture in yogurt production [36]. However, studies have shown that a wide variety of LAB have been isolated. Aslam and Qazi (2010) isolated *Lactobacillus delbrueckii* subsp. *bulgaricus*, *Lacticaseibacillus casei*, *Lactobacillus acidophilus* and *Ligilactobacillus salivarius* from local yogurts in their study and reported that they have high acid tolerance properties [37]. Similarly, *Latilactobacillus sakei* is a species of microorganism from the same family, which is a facultative heterofermentative that can produce alcohol or lactic acid from sugars. It is used as a starter culture in meat products [38]. For instance, the genus *Lactobacillus*, which is frequently found in meat and processed food, as well as different fermented food products, includes the species *Lactiplantibacillus plantarum*. Sauerkraut, pickles, pickled olives, Korean kimchi, Nigerian ogis, sourdough and some cheeses, fermented sausages, and pickled olives contain *Lactiplantibacillus plantarum*. A homofermentative genus of LAB is called *Lactococcus*. They can be modified by changing environmental factors such as pH, glucose concentration, and nutrient limitations due to their homofermentative nature. They are a Gram-positive, catalase-negative, dormant cocci in single, double or chain forms [9]. These organisms are widely used in the dairy industry in the production of fermented milk products such as cheese. They can be used in starter cultures with single strains or mixed strains with other LAB, such as *Lactobacillus* and *Streptococcus*. *Lactococcus lactis* and its subspecies lactis and cremoris are important starter cultures in industrial dairy fermentation [39].

Another LAB, the *Weissella* species, has been isolated from a wide range of habitats, including milk, vegetables, and different fermented foods such as European sourdoughs and traditional Asian and African fermented foods [9]. Important species of acidophilic and aciduric LAB, along with the food sources from which they were obtained, are shown in Table 1.

### 3.2. Food Safety and Stability Issues 

In traditional fermented food production, the food is produced by inoculating the fermented food that is produced beforehand. However, this situation is mostly valid for home-type food production at present. One of the important steps to ensure food safety and stabilization in large-scale, standard fermented food production is the use of starter cultures. Acidophilic–aciduric microorganisms are also involved in various production processes for fermented foods. In this situation, acidophilic LAB are used as a starter culture for several purposes. Producing fermented dairy products such as meat, fish, fruit, vegetable, and cereal products is the major objective of the usage of acidophilic LAB. Another objective is to enhance the nutritional value, flavor, and texture of fermented foods by producing flavor compounds. In this regard, the maturation process is very significant. The most prevalent acidophilic LAB identified in ripened cheeses are *Pediococcus acidilactici* and *Pediococcus pentosaceus* [41]. Despite not being a starter, acidophilic LAB can be isolated from many foods. For example, nonstarter *Lacticaseibacillus casei*, which has a wide pH and temperature range, can be found in ripe cheddar cheese and Sicilian green olives [9].

In addition, another purpose of the use of acidophilic LAB, which is used as a starter culture, is the support of food hygiene. The majority of acidophilic LAB live independently of any host species, are not pathogenic, and produce no products that are toxic or unpleasant to humans. Therefore, people can consume fermented foods formed by the growth of acidophilic LAB. The reducing conditions provided by these bacteria and low-pH habitats with high concentrations of fermented acids can inhibit the growth of many bacteria [32,42]. For example, they have been reported to inhibit the growth of *Listeria monocytogenes* and *Escherichia coli* O157:H7 in the processed meat products from which *Latilactobacillus sakei* is isolated [38].

It is clear that the fermentation process used by acidophilic LAB improves food flavor, aroma, and hygiene. It should be noted that some acidophilic and aciduric microorganisms contribute to food spoilage. *Bacillus coagulans*, a microaerophilic, slightly acidophilic, and heat-tolerant species, was isolated from spoiled canned milk and first described in 1915. This microorganism is highly suitable for development in acidic food due to its acidophilic nature. Moreover, it has been isolated from this type of food and is usually identified as the cause of spoilage in dairy products, vegetables, and fruits. High levels of lactic acid are produced as a result of this type of degradation [43]. *Bacillus coagulans* is also a thermophile, but differs from *Geobacillus stearothermophilus* (previously *Bacillus stearothermophilus*) as it can grow at pH values below 4.0 [44]. During deterioration, it causes the product to become tasteless and sour. Generally speaking, *Bacillus coagulans* has caused significant economic losses for the food industry due to flat-sour spoilage in canned foods [45]. Similarly, *Alicyclobacillus acidoterrestris* is an obligate acidophilic that grows optimally at pH 3.5–4.0 and has a pH of 2.5–5.5 for growth. *Alicyclobacillus* is the only spore-producing acidophilic genus that has been described as a cause of spoilage at these pH values to date [9]. *Alicyclobacillus acidoterrestris* spores are generally more heat resistant than other acidophilic spore formers and cause the spoilage of processed fruit and vegetable juices and concentrates [46,47]. Some species in the genus *Alicyclobacillus* have greater perishability as they can produce large amounts of guaiacol, which negatively affects the odor of the product [47]. In addition, butyric anaerobes, *Clostridium butyricum*, *Clostridium beijerinckii* and *Clostridium pasteurianum*, which cause spoilage in low-acid canned foods, are generally associated with the spoilage of products with pH values between 3.9 and 4.5 [9].

In conclusion, acidophilic microorganisms can often result in losses, food deterioration, and changes in food stability. These different acidophilic and aciduric microorganism states occupy different places on the benefit–harm axis. The importance of using acidophilic LAB as a starter culture in the production of fermented foods, in the development of new foods, and in the formation of the texture, taste, aroma and odor of products, is indisputable. Moreover, they improve food hygiene by limiting other microbial species’ ability to reproduce by balancing the pH of the food. In acidic food production lines, where production hygiene and processes are taken into consideration, the hazards produced by acidophilic LAB can be minimized, ensuring that the negative impacts of these microorganisms on food safety and stability do not occur.

### 3.3. Effects of Acidophilic Lactic Acid Bacteria on the Nutritional Value of Foods

It is known that acidophilic LAB are generally used in the production of fermented products. In this regard, the expected benefits and effects of the fermentation process can often be evaluated as the benefits provided by acidophilic LAB.

The most important output of the fermentation process is the production of organic acids. These organic acids (lactic acid, acetic acid, formic acid, propionic acid) are important food preservatives [48]. The antagonistic activity of acidophilic LAB against other microorganisms is due to the metabolites they produce, such as bacteriocin [49]. Thus, acidophilic LAB provide hygienic conditions by preventing and even eliminating food pathogens [40], since acidophilic LAB are used as bioprotective cultures [50]. Acidophilic LAB provide hygienic and organoleptic benefits, especially in fermented foods such as yogurt, wine and cheese [28]. In addition to its known benefits, acidophilic LAB can bind heavy metals in the water and environmental matrix. This is especially useful in the aquaculture industry [48].

Moreover, acidophilic LAB are used in the production of probiotic foods, which plays a critical role in maintaining human health. Due to their potential benefits as probiotics, the acidophilic *Lactobacillus* species are the most commonly used group of microorganisms within the LAB group [51]. Acidophilic LAB are also important because of the antimicrobial and antifungal substances they produce. Thus, they also reduce the formation of mycotoxins. In addition, it is possible to increase the bioavailability of grain-based products by fermentation with the fermentation activities of LAB [52]. It is possible to obtain healthier, more delicious and innovative products with the lactic acid fermentation of legumes [53]. The benefits of the fermented product produced by acidophilic LAB fermentation of soy milk, whose consumption has increased in recent years, are also emphasized. Various enhanced health benefits of this fermented product have also been reported, including bioactive compounds, enhanced nutritional values, and antihypertensive, antioxidant, antidiabetic, anticancer, and hypocholesterolemic effects [54]. It was also reported that the soluble dietary fiber, total polyphenol content and organic acid levels of potatoes increased at the end of the fermentation process of sweet potatoes using acidophilic LAB [55]. It is also known that many intestinal and urinary pathogenic bacteria are inhibited by the antagonistic activity of these microorganisms [37]. Furthermore, certain strains of the important acidophilus *Lactobacillus acidophilus* have been found to absorb cholesterol in the intestines [51]. It has also been reported that LAB’s dominance in the gut microbiota may be one of the ways to treat obesity [56].

**Figure 1:**
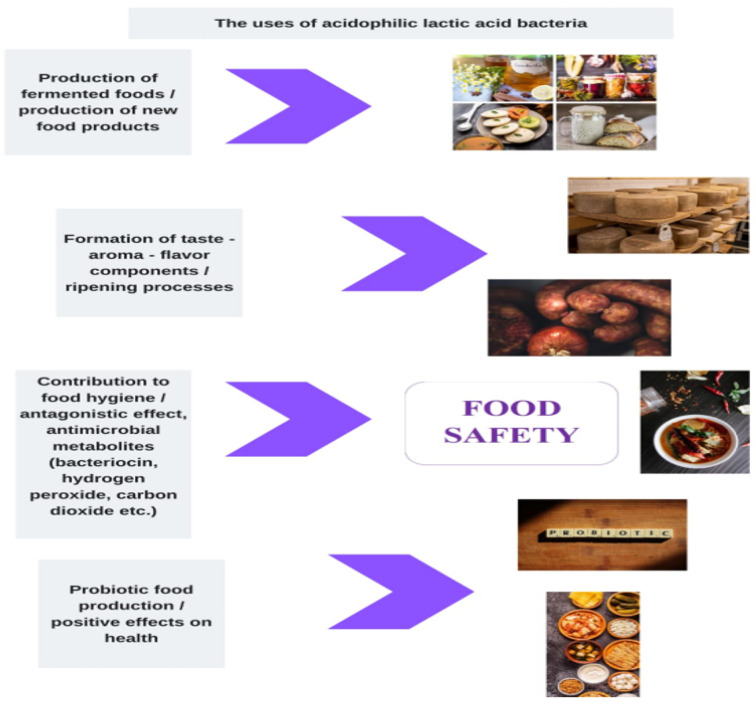
The effects of low-pH microorganisms on the nutritional value and safety of foods.

In general, acidophilic–aciduric microorganisms affect the nutritional value of foods, both by preserving the food and its positive effects on human health. Specifically, with the use of acidophilic LAB as a starter culture in foods, it is stated that aroma, taste, texture, odor, and beneficial microorganism growth increase, while preservatives, artificial sweeteners, sucrose, lactose, oil and contaminants decrease in food [31]. Although many food-preserving processes and substances have been developed in advanced food production techniques, the conditions provided by fermentation are essential for ensuring the shelf life and microbiological safety of products [33]. Figure 1 shows the effects of low-pH microorganisms on the nutritional values and safety of foods.

## 4. Acidophilic Lactic Acid Bacteria’s Role in the Human Diet and Health

### 4.1. Their Probiotic Potentials

Live microorganisms that, when provided to a host in sufficient quantities, confer a health benefit to the host are known as probiotics [57]. Relationships with the health benefits of probiotics, which have been in human’s lives since fermented dairy products were first consumed, only date to the beginning of the last century. Over the years, the understanding of the health benefits of probiotics and the identification of microorganisms with probiotic potential have encouraged consumers and the food industry to use these microorganisms worldwide [58,59]. The global market size for probiotics was estimated to be USD 54 billion in 2020 and will reach USD 111.21 billion by 2030 [60].

Inhibition of the growth of pathogenic organisms such as *Salmonella*, *Shigella*, and *Helicobacter*, prevention/reduction of diarrhea and lactose intolerance symptoms, support of immunity, modulation of gut microbiota, immunomodulation, reduction in serum ammonia and cholesterol levels, anticarcinogenic and antimutagenic activities, and alleviation of allergies and atopic diseases are among the health benefits of low-pH probiotics [4,5,6]. As a result, the probiotic industries are exerting a significant amount of effort to find new probiotic strains that are effective, promising, and efficient, and that have a positive impact on the health of consumers [61]. 

For microorganisms to possess probiotic properties, they must be able to traverse the gastrointestinal tract, endure the acidic conditions of the stomach, and be resistant to bile salts [5,62]. They need to stick to the intestinal mucosa and establish a short-term colony in the colon [5,63,64]. Simultaneously, probiotic food products must contain an adequate amount of live probiotic microorganisms [5]. 

The most common probiotics microorganisms belong to the genus *Lactobacillus*, *Leuconostoc*, *Pediococcus*, and *Bifidobacterium* [65,66]. Since LAB are native inhabitants of the healthy human gut and are also present in many dairy products and conventional fermented foods, they are thought to be the most suitable acidophilic candidates for probiotics, aside from pathogenic strains [61]. LAB are generally regarded as potential probiotic bacteria that are prevalent in nature and have a wide range of applications in the food industry [6]. The main types of LAB with probiotic properties are *Lactobacillus acidophilus*, *Lacticaseibacillus casei*, *Lactobacillus johnsonii*, *Lacticaseibacillus rhamnosus*, *Limosilactobacillus reuteri*, *Lactiplantibacillus plantarum*, *Limosilactobacillus fermentum* and *Lactiplantibacillus pentosus* [5,67]. Being resistant to a low pH, bile salt, pepsin, and pancreatin, *Pediococcus pentosaceus* OZF is one of the acidophilic forms of LAB that can survive in the upper part of the gastrointestinal tract, and thus has a potential probiotic effect on the host organism [68]. Although LAB are mainly isolated from dairy products, they can also be obtained from different fermented foods, such as fermented meats and vegetables [5]. The isolation, identification, and characterization of the new acidophilic LAB has two main benefits, identifying the characteristic taxonomy of these microorganisms and revealing promising useful and functional new probiotic microorganisms [6,69]. 

Plessas et al. (2017) evaluated the probiotic potential of 45 LAB strains isolated from feta-type cheese by subjecting them to a series of tests, including resistance to low pH, resistance to pepsin and pancreatin, and tolerance of bile salts. It was determined that the strain defined as *Lacticaseibacillus paracasei* K5 has a similar or even better/more desirable probiotic properties than the reference strain *Lactiplantibacillus plantarum* ATCC 14917 [5]. *Enterococcus durum* LAB18S is another acidophilic LAB isolated from cheese that shows probiotic properties [70]. The *Lactococcus hircilactis* strain CH4, *Lactobacillus delbrueckii* strain GRIPUMSK, *Lactobacillus johnsonii* strain PUMSKGRI, and *Lactobacillus leichmannii* strain SKGRIPUM were determined as acidophilic LAB with the highest probiotic properties among 41 bacterial isolates obtained from homemade fermented food products (cheese, curd, fermented rice water, yogurt, and buttermilk) [71]. Falfán-Cortés et al. (2022) found that *Lacticaseibacillus paracasei* has the highest probiotic potential among the acidophilic LAB isolated from tenate cheese [72]. It is also reported that yogurt made from Yak milk contains higher amounts of probiotic LAB than yogurt made from other bovine milk, and acidophilic *Limosilactobacillus fermentum* (31%) and *Lacticaseibacillus casei* (28%), noted to be probiotics, are predominant [73,74]. 

The *Lactobacillus gasseri* MA-4 strain was observed to have a high tolerance to a low pH, bile salts, artificial gastric and small intestinal fluids, and was isolated from human breast milk. This strain was found to be a suitable candidate for use as a probiotic according to the findings of a study that evaluated the probiotic potential of this acidophilic strain [10]. *Lactobacillus gasseri*, one of the dominant species of the *Lactobacillus* group, is one of the low-pH probiotic microorganisms with a Generally Recognized As Safe (GRAS) status [75]. Another study concluded that 17 LAB isolated from yak milk also showed excellent probiotic properties, along with desirable health benefits [61]. In one of two different studies, *Lactiplantibacillus plantarum*, *Lactococcus lactis*, and *Enterococcus lactis* were found to be effective probiotics among all LAB obtained from camel milk [76], while in the other, *Bifidobacterium longum* obtained from camel milk was concluded to be the best potential probiotic [77]. In Nigerian goat milk, only six (*Weissella cibaria* GM 93m3, *Weissella confusa* GM 92m1, *Pediococcus acidilactici* GM 18a, *Pediococcus pentosaceus* GM 23d, *Lactiplantibacillus pentosus* GM 102s4 and *Limosilactobacillus fermentum* GM 30m1) of the 27 evaluated LAB were found to survive in pH 2.5, 0.3% bile salt, stimulated gastrointestinal conditions, and had auto-aggregative and hydrophobic properties [78]. 

The increasing global interest in products with functional properties has encouraged the search for new acidophilic microorganisms, especially the acidophilic LAB found in natural sources such as traditional fermented foods [6,11]. Many traditional fermented foods, especially fermented milk products, are accepted as vital sources of acidophilic LAB that are beneficial to human health [79]. LAB are food-fermentation agents used in the production of numerous foods, including yogurt, cheese, cultured butter, sour cream, sausages, cucumber pickles, olives, sauerkraut, and cocoa [80]. Revealing the probiotic potential of acidophilic LAB will help to determine which of these microorganisms can be used for probiotic purposes, especially in the health and food industry. Studies investigating the probiotic potentials of acidophilic and aciduric LAB isolated from some traditional fermented foods are demonstrated in Table 2.

### 4.2. Beneficial and Toxic Compounds Released by Acidophilic Lactic Acid Bacteria

**Figure 2:**
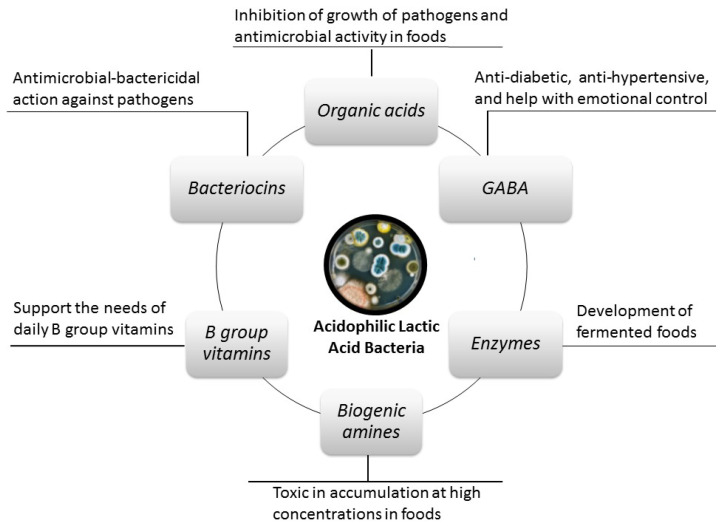
Some compounds released by acidophilic lactic acid bacteria and their health effects.

Although some acidophilic lactic acid bacteria and traditional fermented foods are associated with many beneficial effects on human health, the presence of some toxic compounds released by these microorganisms is also known. The beneficial effects mentioned in the literature are mostly attributed to the beneficial compounds released by these acidophilic LAB (Figure 2) [10,11,12]. Of these compounds, organic acids, some B group vitamins, GABA, amylase enzyme, and bacteriocins have important therapeutic-beneficial effects on host health [10,11,12], while biogenic amines (BA) are not desirable compounds (Figure 2) [13,14].

#### 4.2.1. Organic Acids

Organic acids released from acidophilic LAB show antimicrobial activity [90]. LAB produce a wide variety of organic acids, such as acetic acid, lactic acid, benzoic acid, sorbic acid, formic acid, citric acid, succinic acid, and propionic acid, as the end product of the fermentation of carbohydrates [90,91]. Organic acids, predominantly synthesized from acidophilic LAB, such as lactic acid, benzoic acid, and sorbic acid, create an unfavorable environment for the growth of pathogenic microorganisms [91]. Wang et al. discovered that 0.5% lactic acid inhibits the growth of pathogens, including *Salmonella enteritidis*, *Escherichia coli*, and *Listeria monocytogenes* [92]. Benzoic acid alone inhibits the growth of *Enterobacter agglomerans* by 10 to 15%; when combined with lactic acid, it can inhibit growth by up to 100% [90]. Salomskiene et al. (2019) determined that the LAB strains produced organic acids, which are natural antimicrobial compounds such as ethanol, lactic, citric, benzoic, and sorbic acids. They also reported that *Lactobacillus helveticus* was the best producer of benzoic acid [90]. LAB such as *Lactobacillus*, *Lactococcus*, *Leuconostoc*, *Streptococcus*, and *Pediococcus* are known as starter cultures frequently used for the fermentation of milk, meat, and vegetable products that produce organic acids as the final products [91]. In light of these, it is possible to hypothesize that LAB can maintain antimicrobial activity by producing organic acids like sorbic and benzoic acids in significant quantities that inhibit food pathogens, particularly in fermented foods. New industrial-based studies should be planned, though, as the effective production of organic acids on an industrial scale has not yet been accomplished.

#### 4.2.2. B Group Vitamins

For every living cell, vitamins are essential micronutrients. They perform as precursors or as participants in a variety of key enzymatic processes, including electron transport chains. Humans are unable to produce the B-group vitamins and must therefore rely on external sources to meet their daily needs, in contrast to microorganisms, which can typically biosynthesize them according to their needs [93]. Vitamin deficiencies can still occur in many countries, despite the fact that the majority of vitamins can be found in a wide variety of foods. This is primarily caused by insufficient food intake and/or an unbalanced diet [94,95]. In recent years, the growing interest in fortifying foods with vitamins of microbial origin has led scientists to focus on identifying LAB strains that are GRAS and capable of synthesizing essential vitamins and other biomolecules [93,96]. It is known that some LAB strains can produce/release and/or increase some B group vitamins such as thiamine (B_1_), riboflavin (B_2_), pyridoxine (B_6_), folate (B_9_), and cobalamin (B_12_) [95,97]. Numerous acidophilic LAB species, including *Lactococcus lactis*, *Lactobacillus gasseri*, and *Limosilactobacillus reuteri*, have been suggested to play a part in the production of vitamins [93]. Hati et al. (2019) found out that *Lactiplantibacillus plantarum* produced the highest B_2_ and *Limosilactobacillus fermentum* produced the highest B_12_ and folate among the acidophilic *Lactobacillus* isolates they isolated from traditional Indian fermented foods [93]. In another study, it was determined that *Limosilactobacillus reuteri* JCM1112 produces vitamins B_12_ and folate [98]. Two strains of *Latilactobacillus sakei* and *Lactiplantibacillus plantarum* were found to produce high levels of folate in a study that evaluated the folate, vitamin B_12_ and B_1_ production of LAB isolated from nukazuke, a traditional Japanese pickle. However, the study did not find any strains of LAB that produced high levels of vitamin B_12_ and B_1_ [99]. Increased serum B_2_ and B_9_ levels were observed in mouse models that consumed pasta made with quinoa sourdough fermented with *Lactiplantibacillus plantarum* strains, a acidophilic LAB that produces vitamins B_2_, B_9_, and phytase [100]. In the food industries, LAB can be used to increase the concentrations of group B vitamins (especially folate) in yogurts [101,102]. Laiño et al. (2013) discovered that yogurt prepared with folate-producing *Lactobacillus delbrueckii* subsp. *bulgaricus* and *Streptococcus thermophilus* strains had a statistically significantly higher folate content [101]. Soybean-based functional food production using acidophilic LAB capable of producing compounds with B_12_ activity is also seen as an alternative method to prevent vitamin deficiency [103]. Moreover, there are genetic engineering efforts to increase the production of B group vitamins or to create new vitamin-producing strains [95,104]. However, the use of genetic engineering for this purpose should be discussed in a broader perspective. As a result, it can be thought that the bio-enrichment of foods (such as milk, cereals, and derivatives) using acidophilic LAB with proven effectiveness may be a new strategy to increase the bioavailability of vitamins in foods. In addition, it is predicted that the use of vitamin-producing LAB in the food industry can be a cost-effective alternative to existing vitamin supplementation programs.

#### 4.2.3. Gamma-Aminobutyric Acid (GABA)

Some acidophilic LAB can produce GABA bioactive compounds. GABA is produced by the conversion of glutamic acid with the enzyme glutamate decarboxylase. GABA has functions such as primary neurotransmission inhibitor, diabetic suppressor and antihypertensive, and helps with emotional control in the sympathetic nervous system [105]. In addition, it is thought that the use of GABA-enriched foods may provide health benefits due to the regulation of depression, insomnia, and autonomic disorders, and their anti-diabetic effects [106]. Many GABA-enriched functional foods are reported to be developed, including fermented seaweed drinks, black raspberry juice, and dairy products [106,107,108]. Increasing GABA production in a seaweed beverage fermented with acidophilic *Lactiplantibacillus plantarum* DW12 as a starter culture [107], and developing yogurt containing high levels of GABA, free amino acids, and isoflavones using LAB and germinated soybean extract, are examples of these enrichments [108]. In a study in which *Lactobacillus namurensis* NH2 and *Pediococcus pentosaceus* HN8 were used to increase the GABA content in Thai fermented pork sausage, it was found that the GABA content of the sausages increased eight times after the intervention, and these GABA-enriched sausages had a better taste and lower fat, carbohydrate, and energy contents than controls [109]. Jitpakdee et al. (2021) reported *Pediococcus pentosaceus* ENM104 and *Lactiplantibacillus plantarum* SPS109 isolated from Thai fermented foods as GABA-producing low-pH probiotic candidates [105]. Lozano et al. (2022) found that, among 101 Lactobacillus strains isolated from natural whey starters used by Uruguayan artisans in cheese production, 15 strains from the *Lactiplantibacillus* group had a significantly higher GABA production than the others [110]. *Lactobacillus* and *Lactococcus* spp. are also acidophilic LAB that were previously reported to produce GABA [106,108,111]. Considering the potential health benefits, it is crucial to define the complete list of acidophilic LAB that produce high levels of GABA, with new studies to be carried out. The fact that functional foods are increasingly preferred by consumers over the years suggests that the use of these GABA-producing microorganisms in the food industry will become widespread.

#### 4.2.4. Enzymes

Some LAB also produce enzymes such as proteases, peptidases, polysaccharide degrading enzymes, lipases, amylases, esterases, and phenoloxidases [12]. One of the acidophilic LAB, *Lactobacillus acidophilus* SAM1, produces lipase enzymes, whereas *Lactiplantabacillus plantarum* SAM2 produces amylase and protease enzymes [63]. Some acidophilic LAB may also help reduce the risk of lactose intolerance by producing the lactase enzyme, which breaks down lactose into simple sugars [1,112]. Amylolytic LAB mainly belongs to the genera *Lactobacillus*, *Lactococcus*, *Streptococcus*, *Pediococcus*, *Carnobacterium*, and *Weissella* [113]. Tallapragada et al. (2018) came to the conclusion that the acidophilic–aciduric *Lactobacillus* species *Limosilactobacillus fermentum* and *Lactobacillus sp* G3_4_1TO2 produce amylase enzyme, and that *Lactobacillus* spp. G3_4_1TO2 from these two bacteria is a potential probiotic bacterium that produces maximum amylase enzyme [12]. Another study showed that 132 of the LAB found in fermented grain products in China produced amylase, and the *Lactiplantibacillus plantarum* strain produced the most [114]. Other acidophilic LAB with amylolytic activity (by making the enzyme amylase) include *Lactobacillus amylovorus* and *Lacticaseibacillus manihotivorans* [12,113]. Amylases catalyze the initial hydrolysis of starch to short oligosaccharides [115]. The use of amylolactic acid bacteria in conjunction with starch results in an improved fermentation process that is more efficient and cost-effective [12]. Because microbial amylases are more stable than plant and animal amylases, and because they are simpler and more affordable to manipulate, they can be used to produce enzymes with the desired properties in large quantities [116,117]. In light of these studies, the use of amylolactic acid bacteria in the development of grain-based foods and fermented foods/beverages can be recommended. 

#### 4.2.5. Bacteriocins

Bacteriocins are ribosomally synthesized antimicrobial peptides produced by certain microorganisms, including LAB, that are active against closely related microorganism, mostly Gram-positive bacteria, to obtain a competitive advantage for nutrients in the environment [118,119]. They usually have bactericidal and/or bacteriostatic activity targeting the bacterial cytoplasmic membrane [120]. Bacteriocins are divided into four main classes [118]. The first group of bacteriocins is generally known as lantibiotics. Nisin, one of the most commonly used and studied bacteriocins, can be provided as an example of this group of bacteriocins. Examples of bacteriocins in the second group are Pediocin PA1, Lactococcin A and B, Leucocin A, Sakacins A and P, Curvacin A, and Bavaricin MN, which are heat-resistant and hydrophobic, and inhibit *Listeria monocytogenes* [1,118]. The third group of bacteriocins is larger than 30 kDa and are heat-stable peptides (for instance, helveticin J), while the fourth group of bacteriocins is classified as bacteriolysins, that is, hydrolytic polypeptides [1,118].

The use of antibiotics as a growth-promoter in animal husbandry and aquaculture has been banned in various countries due to the increase in antibiotic resistance worldwide [121]. The use of antimicrobial growth-promoters (AGPs) in animal production has been discouraged in some nations, including the United States, Canada, and Japan [121,122]. However, the livestock industry is negatively impacted by the ban on using antibiotics as AGPs owing to various, uncontrolled bacterial diseases [121,123]. Because of this, it has become necessary to find new non-antibiotic growth-promoters in order to enhance animal growth and reproduction, as well as control multiple bacterial infections. Probiotics are one of the potential non-antibiotic growth-promoters being researched for this purpose [121]. Acidophilic LAB are potential probiotics being evaluated as an alternative to antibiotics during food–animal production [121]. Bacteriocins synthesized from LAB ribosomes are the antimicrobial peptides primarily responsible for this effect [124]. A large number of the bacteriocins made by LAB are secure and effective against a specific or broad range of bacteria. They may also possess sporostatic or sporicidal properties toward bacterial spores [124,125]. For instance, laparaxin is an antibacterial polypeptide secreted by *Lacticaseibacillus paracasei* NRRL B-50314, which has antibacterial activity against numerous Gram-positive bacteria [124,126]. It can be concluded that some bacteriocins released by LAB show potential as antibacterial agents and as new non-antibiotic growth-promoters. In addition, the rapidly rising antibiotic resistance worldwide suggests that LAB and bacteriocins may be the only treatment for some clinical cases in future and/or may need to be combined with low-dose antibiotics. However, to reach definite conclusions, it is necessary to determine from which bacteria antibacterial bacteriocins are produced and to evaluate the bactericidal effect of these bacteriocins with new studies.

Bacteriocins released by some acidophilic LAB also attract attention by their potential to reduce the growth of pathogenic microorganisms in foods, as well as being seen as potential non-antibiotic new growth-promoters in livestock [127,128]. The fact that most bacteriocins are generally colorless, odorless, and tasteless makes them usable in the food industry [129]. *Lactococcus lactis* bacteria from acidophilic LAB create the polycyclic antibacterial peptide known as lantibiotic nisin. This bacteriocin, the first antimicrobial peptide approved for use as a food preservative, is applied as a broad-spectrum antibacterial agent against many bacteria that are food-spoilage pathogens [124,125,130]. Nisin and pediocin are two bacteriocins approved by the Food and Drug Administration (FDA) for use in the food industry [118]. In order to produce high-quality fermentation products, the dairy industry frequently uses *Lactobacillus acidophilus*, another acidophilic LAB that produces the antimicrobial compound bacteriocin [63]. Additionally, *Pediococcus pentosaceus*, an acidophilic microorganism, creates anti-Listerial bacteriocin [68,127]. Todorov et al. demonstrated that an anti-listerial bacteriocin, Pediocin ST18, is produced by *Pediococcus pentosaceus* isolated from boza, a traditional fermented beverage [127]. Different bacteriocins, such as Leucocin OZ and Leucocin F10, are produced by *Leuconostoc carnosum*, a lactic acid bacterium isolated from fermented meat products [131]. These bacteriocins, produced by acidophilic LAB, show antimicrobial–bactericidal activity against some foodborne pathogenic bacteria, such as *Listeria Monocytogenes* and *Listeria innocua* [127]. In line with the data obtained from the literature, it can be concluded that the bacteriocins released by these acidophilic LAB generally have a bactericidal effect against the bacteria responsible for food spoilage and food-borne pathogens, and their use in the food industry may become widespread in the coming years, along with a clearer understanding of their effects.

The failure of current therapies for *Clostridium difficile* infections and the rise in recurrence has compelled the creation of new ones [130,132]. The use of probiotic bacteria that generate antimicrobial molecules, such as bacteriocins, has recently emerged as a promising alternative for the prevention and treatment of diseases linked to *Clostridium difficile* [130]. Even though numerous bacteriocins, including Nisin, Microbisporicin, Lacticin 3147, and thuricin CD, are effective against Clostridium difficile, only Nisin has been recognized as a natural food additive by the FDA, WHO, and the European Union to date [130,133]. It is stated that nisin released by the acidophilic *Lactococcus lactis* can be used to control *Clostridium difficile* infections. [133,134]. However, Le Lay et al. (2015) discovered that, in a human colon model, nisin Z produced by *Lactococcus lactis* UL719 did not inhibit *Clostridium difficile* [130]. The use of these acidophilic LAB, which positively affects some diseases such as *Clostridium difficile* infections, and the bacteriocins they produce, such as nisin, can be considered in the treatment process. However, there are very few and contradictory study results in the literature, making them difficult to interpret. 

Bacteriocins produced by acidophilic LAB are being researched as a promising antiviral alternative to traditional antiviral agents, in addition to their antibacterial activities. The antiviral activity of bacteriocins, which has previously received much less attention than their antibacterial activity, is currently the subject of extensive research [129]. Bacteriocins, which are mainly produced from probiotic bacteria, can reduce the viral load of the host and improve the immunomodulatory mechanism against viral infections [1,129]. It is reported that some bacteriocins produced by some acidophilic LAB hace anti-influenza virus activity [135]. Maeda et al. also determined that *Lactiplantibacillus plantarum* L-137 from LAB presented with activity against the influenza virus [136]. The *Enterococcus faecium*-produced Enterocin ST5HA, *Enterococcus faecalis*-produced Enterocin AAR-74 and Enterococcus AAR-71, and *Enterococcus mundtii*-produced Enterocin CRL35 and Enterococcus ST4V from acidophilic LAB are additional examples of known antiviral bacteriocins [1,129]. Enterocin ST4V and enterocin CRL35 are reported to inhibit herpes simplex virus (HSV) types 1 and 2 in a dose-dependent manner, mainly by inhibiting late glycoprotein synthesis [129]. These data indicate that bacteriocins released from acidophilic LAB with antiviral activities can also be used as antiviral agents in the prevention and treatment of viral infections. Although the detailed mechanism(s) for the antiviral activities of acidophilic LAB has not yet been fully elucidated, the scientific community is increasingly focusing on the benefits of these microorganisms and their metabolites in the fight against viruses.

#### 4.2.6. Biogenic Amines

One of the undesirable compounds released from acidophilic LAB is BAs. BAs are low-molecular-weight nitrogenous organic bases that can be accumulated in foods at high concentrations due to their microbial activity and have toxic effects on consumers [14]. LAB are believed to be the primary producers of BAs in fermented foods [13]. In the meantime, the leading producers of BAs in dairy products are mostly LAB belonging to the *Enterococcus*, *Lactobacillus*, *Leuconostoc*, *Lactococcus*, and *Streptococcus* genera [137]. In a study testing the biogenic amine production of fifteen LAB strains, *Lacticaseibacillus casei* (TISTR 389) and *Lactobacillus delbrueckii* subsp. *bulgaricus* (TISTR 895) were found to be potentially acidophilic microorganisms for the production of histamine and tyramine. In another study, the TISTR 389 strain produced the highest levels of histamine and tyramine [138]. Foods likely to contain high levels of biogenic amines include fish, fish products, and fermented foods (meat, dairy products, some vegetables, beers, and wines) [14]. The consumption of foods or beverages containing high concentrations of BA is a risk factor for consumer health because it may cause symptoms including flushing, headache, heart palpitations, vomiting, diarrhea, and an increase or decrease in blood pressure [13,14]. Additionally, they can cause the organoleptic properties of the food they are in to depreciate [14]. However, their toxic effects depend on the type of BA, individual susceptibility, allergy, and consumption of monoamine oxidase inhibitor drugs or ethanol, which interact with the amino oxidase enzymatic systems responsible for the detoxification process of exogenous BAs [13]. 

Histamine, tyramine, putrescine, cadaverine, and phenylethylamine are the most important BAs found in food and are generated by the decarboxylation histidine, tyrosine, ornithine, lysine, and phenylalanine, respectively [14]. Histamine and tyramine are regarded as the most dangerous BAs due to the severity of the symptoms they can cause [13]. Putrescine, cadaverine, phenylethylamine, agmatine, spermine, and spermidine may be toxic, but they may also make histamine and tyramine toxicity worse by inhibiting the enzymes that break down these substances down [13,139]. Since tyramine is mostly found in cheese, tyramine poisoning is known as the “cheese reaction”. Tyramine poisoning can cause diet-induced migraine, increased cardiac output, nausea, vomiting, respiratory disorders, and high blood sugar [13,140]. There is no specific legislation regarding the presence of BAs in food, with the exception of fishery products, where the maximum acceptable level of histamine is set, even though eating foods high in BA can have toxicological effects [13]. However, a qualitative risk assessment of BA in fermented foods by EFSA has revealed concentrations that could have negative effects on consumers. According to this report, in order to lower the risk of BA toxicity in foods, hygienic precautions, additional microbial controls, and the use of starter cultures that do not produce BA are crucial [141]. Moreover, to prevent BA toxicity, the potential risk can be evaluated by analyzing the BA-forming ability of the bacteria for use in fermentation [140]. However, more in vitro and in vivo research on BA and its toxicities is needed.

### 4.3. Safety Assessments of Acidophilic LAB with Probiotic Potentials

**Figure 3:**
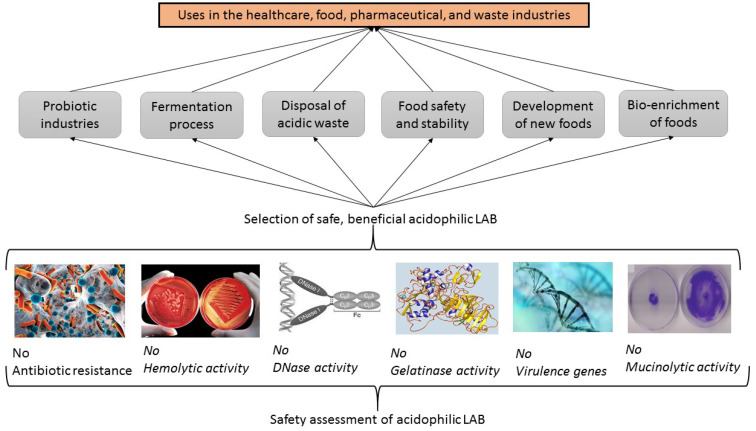
Safety assessments of acidophilic lactic acid bacteria (LAB) with probiotic potential.

Microorganisms with probiotic potential can be used for the improvement of dysbiosis, which is an imbalance in the enteric microbial population of the intestinal microbiota, with many different health effects [10]. However, before all microorganisms, including acidophilic LAB, can be used for their probiotic potential, their safety must be evaluated and verified for the food and human health industries with some activity tests (Figure 3) [5,17].

#### 4.3.1. Antibiotic Resistance

Microorganisms, especially probiotics, should not spread antibiotic-resistance genes since they may interact with the normal microbiota of the human gastrointestinal tract after consumption [19]. Although the majority of probiotic bacteria have been granted GRAS status, there is still cause for concern regarding the potential transfer of antibiotic-resistance genes from used probiotics, including some LAB [5,6,17]. Over the years, the development of resistance to antibiotics has turned into a global public health problem [142]. Although the development of resistance to antibiotics has mostly been investigated on pathogenic microorganisms, some recent studies examine the ability of LAB to develop resistance to antibiotics such as tetracycline, erythromycin, and vancomycin [142]. In a study evaluating the antibiotic resistance of *Lactobacillus leichmannii*, *Lacticaseibacillus casei*, *Lactobacillus delbrueckii*, *Levilactobacillus brevis*, *Limosilactobacillus fermentum*, *Lactobacillus coagulans*, *Lactobacillus acidophilus*, *Lactobacillus lactis*, and *Lacticaseibacillus rhamnosus* strains, it was found that *Lacticaseibacillus casei*, *Lactobacillus delbrueckii*, and *Levilactobacillus brevis* strains had a higher resistance to antibiotics [18]. It has also been reported that some strains of *Enterococcus faecium*, one of the acidophilic LAB, can transfer vancomycin-resistance genes to *Lactobacillus acidophilus* [142]. Many people who suffer from common bacterial infections that were once easily treated with antibiotics may experience serious health issues as a result of possible antibiotic resistance [17]. EFSA has advised that screening for antibiotic resistance in potential probiotics should become mandatory [143]. For this reason, it can be said that acidophilic LAB, whose probiotic properties are mentioned in this review, should be screened for antibiotic resistance before using them for probiotic purposes.

#### 4.3.2. Hemolytic Activity

It is important that acidophilic LAB with probiotic qualities should not show hemolytic activity in order to be used safely [5,63,64,144]. Non-hemolytic activity is considered a safety prerequisite for the selection of probiotic strains [18]. Even if bacteria have GRAS or Quality Presumption of Safety (QPS) status, EFSA strongly advises investigating their hemolytic activities [77]. Following incubation on Columbia agar plates, the hemolytic activity of the isolated strains is evaluated and categorized in accordance with the lysis of red blood cells in the medium surrounding the colonies [77]. Strains forming green zones on agar plates are classified as α-hemolysis (partial hemolytic), clean zones as β-hemolysis (complete hemolytic), and no zones as γ-hemolysis (non-hemolytic) [145,146]. Only strains with γ-hemolysis are considered safe [145]. In a study evaluating nine probiotic LAB (*Lactobacillus leichmannii*, *Lacticaseibacillus casei*, *Lactobacillus delbrueckii*, *Levilactobacillus brevis*, *Limosilactobacillus fermentum*, *Lactobacillus coagulans*, *Lactobacillus acidophilus*, *Lactobacillus delbrueckii* subsp. *lactis*, and *Lacticaseibacillus rhamnosus*) strains, it was reported that none of them showed β-hemolytic activity. It was also revealed that most of them were γ-hemolytic (without hemolysis), and only two strains (*Lactobacillus coagulans* and *Lacticaseibacillus rhamnosus*) showed α-hemolytic activity [18]. Oh et al. (2015) found that six strains of seven acidophilic LAB (*Lactiplantibacillus pentosus* SW02, *Lactiplantibacillus plantarum* subsp. *Plantarum* SW03, *Latilactobacillus sakei* subsp. *Sakeii* SW04, *Lactiplantibacillus plantarum* subsp. *Plantarum* SW06, *Lactiplantibacillus plantarum* subsp. *Plantarum* SW07, *Pedeococcus acidococcus* SW01) had γ-hemolytic (no hemolytic activity), and *Pediococcus pentosaceus* SW01 also showed α-hemolytic activity, which is partial hemolysis [147]. In another study evaluating the hemolytic activity of 71 LAB with probiotic potential isolated from fermented olives, none of the strains showed β-hemolytic activity, while four strains (*Lactiplantibacillus pentosus* B278, B279, B281, and B285) exhibited α-haemolysis [148]. Ismael et al. (2022) reported that only *Enterococcus faecium* out of 56 LAB isolated from fermented milk products showed α-hemolytic activity, while other strains did not [149]. On the contrary, another study found that *Enterococcus faecium* and *Pediococcus acidilactici* strains did not show hemolytic activity [150]. Motey et al. (2021) also observed β-hemolysis in none of the LAB isolated from fermented milk products, while α-hemolysis was observed in 38% of the strains [151]. In conclusion, while acidophilic LAB with probiotic potential generally do not show hemolytic activity (γ-hemolytic), there are some strains that show hemolytic activity (especially α-hemolytic activity). Therefore, it is recommended to evaluate the hemolytic activities of these microorganisms before they are used in the food industry, livestock, and health sector. 

#### 4.3.3. DNase Activity

Apart from the hemolytic activity and antibiotic resistance, another undesirable feature in acidophilic LAB is DNase activity [152]. For this reason, DNase activity should be considered in the safety assessment made before the acidophilic LAB are used [152,153]. In the study by Rodrigues et al. (2021), in which they tested the safety of 93 isolates identified as LAB isolated from fruits, it was concluded that 14 isolates were not safe for use because they had DNase activity [20]. However, Somashekaraiah et al. (2019) found that none of the 75 LAB strains isolated from the traditional Indian fermented beverage Neera showed direct DNase activity [154]. Likewise, in three different studies evaluating the safety of LAB isolated from dairy systems [155], fruit processing residues [153], and fermented grain products [156], it was determined that none of these acidophilic LAB showed DNase activity. It was also reported that *Levilactobacillus brevis* and *Lacticaseibacillus paracasei* acidophilic bacteria isolated from goat milk do not show DNase activity [157]. The DNase activity was not reported in strains belonging to *Lactiplantibacillus plantarum* and *Enterococcus faecalis* [158]. Furthermore, DNase activity was not observed in any of the LAB strains isolated from breast milk [159]. In most of the studies investigating the DNase activity of LAB, it was concluded that these acidophilic bacteria do not show DNase activity and are safe in this regard [155,156,157,158,159]. 

#### 4.3.4. Gelatinase Activity

Gelatinase activity is accepted as a detrimental factor as it can hydrolyze collagen, initiating an inflammatory response [21]. In addition, gelatinase is expressed as a zinc metalloprotease, a lethal factor that hydrolyzes casein, hemoglobin, and other bioactive compounds in bacteria [160]. Because of these possible harmful effects, it is also important to determine the gelatinase activity of acidophilic LAB [161]. Rodrigues et al. (2021) found that 18 of 93 isolates identified as LAB isolated from fruits had positive gelatinase activity [20]. Muñoz-Atienza et al. (2013) found that *Enterococcus faecalis* and *Enterococcus faecium* (71% and 11%, respectively) had gelatinase activity among 99 examined LAB [161]. On the contrary, Sakoui et al. (2022) reported that strains of *Enterococcus* genus (*Enterococcus faecium* V6-112, *Enterococcus faecalis* JM102, *Enterococcus mundtii* MAV6B, *Enterococcus gallinarum* HBUAS52471, *Enterococcus casseflavus* APHG2) did not show gelatinase activity [21]. Ben Farhat et al. (2022) also did not observe gelatinase activity in any of the LAB strains of *Limosilactobacillus fermentum* (4 strains), *Lacticaseibacillus paracasei* (1 strain), *Lacticaseibacillus rhamnosus* (1 strain) [162]. In another study, it was revealed that strains belonging to the *Lactiplantibacillus plantarum* and *Enterococcus faecalis* groups did not show gelatinase activities [158]. *Limosilactobacillus fermentum* KL4 and *Lactiplantibacillus plantarum* MOBL1 strains, which are acidophilic LAB, also do not show gelatinase activity [163]. Kaktcham et al. (2018) reported that none of the seven LAB strains (*Lactococcus lactis* subsp. *lactis* 1FT, 1FW, and 3FT; *Lactiplantibacillus plantarum* 1MTK, 4BC, and 13BC and *Levilactobacillus brevis* 1BT) that they investigated for safety showed gelatinase activity [164]. Based on all these studies, there are concerns about whether some strains from LAB (especially strains belonging to the *Enterococcus* genus) have gelatinase activity. It is becoming increasingly crucial to evaluate the safety of acidophilic LAB that are considered for application in the food industry before use. In recent years, gelatinase activity has been seen as a remarkable determinant of the safety of low-pH microorganisms with known probiotic potential. 

#### 4.3.5. Presence of Virulence Genes

For microorganisms to be considered probiotics, they must not contain virulence genes [22]. For this reason, investigating virulence characteristics in the selection of LAB that are to be used in the food industry is another important safety consideration. Some of the virulence genes were investigated in the following strains: gelatinase (gelE), aggregation substance (asa1), enterococcal surface protein (esp), cytolysin (cylA), endocarditis antigen (efaA), adhesion of collagen (ace) [165]. Ribeiro et al. (2014) found that *Enterococci* from LAB isolated from Pico cheese, a traditional cow’s milk cheese, was positive for the presence of some virulence genes [166]. In another study by Domingos-Lopes et al. (2017), investigating the safety of LAB isolated from traditional Pico cheese, it was found that all *Enterococcus* isolates exhibited at least one virulence gene. In the same study, one *Leuconostoc mesenteroides* (L3C21R7) strain and five *Lacticaseibacillus paracasei* subsp. *paracasei* (L2A1K8, L2B21R1a, L2B21R3, L3B1M2, L3C21M6) strains have no tested virulence genes and these low-pH strains are good candidates to be used safely as starter/helper cultures in food fermentation [165]. Similarly, in a study reporting on safety concerns regarding the presence of virulence genes in the *Enterococcus* genus in 99 LAB, it was concluded that most of the *Enterococcus faecalis* strains (20 strains, 95%) harbor at least one relevant virulence factor (efaAfs (95%), gelE (71%), or agg (67%) genes) [161]. However, in another study investigating the presence of virulence genes (agg, gelE, esp, efaAfs, efaAfm and cylA, cylB, cylM, cylLL, and cylLS) in 280 LAB strains, it was reported that none of the strains harbored virulence genes [22]. Considering the health risks of strains with known virulence genes and the risk of transferring these genes to pathogenic microorganisms, it can be concluded that their application in the pharmaceutical and food industries raises concerns. Therefore, even if acidophilic LAB have GRAS or QPS status, whether they harbor virulence genes should be verified in vivo.

#### 4.3.6. Mucinolytic Activity

The mucus that lines the intestinal epithelium and serves as a home for the commensal flora is composed primarily of mucins [167]. Thus, mucin, a glycosylated protein, provides the first line of defense and prevents the translocation of bacteria [168]. Because the production of mucin-degrading enzymes is a virulence determinant and affects the intestinal mucosal barrier, mucinolytic activity is also considered an undesirable property for microorganisms with probiotic potential (including acidophilic LAB). Another way that pathogens and toxins can enter the host is if an invading organism has mucin-degrading activity [169]. For this reason, the mucinolytic activities of acidophilic LAB are also investigated before they are used for probiotic purposes. Rabaoui et al. (2022) revealed that 15% of 47 analyzed LAB strains showed mucinolytic activity, while mucin degradation was dependent on glucose in 21% of the strains. In the same study, it was observed that 32% of *Levilactobacillus brevis* species and 37% of *Enterococcus* species were able to degrade mucin [167]. It has been reported that the mucin degradation of some LAB strains is nutrient-dependent. These microorganisms do not show mucinolytic activity in the presence of ready-to-use carbohydrates as an energy source [167]. In another study, 11 isolates of 93 LAB isolates were found to have mucinolytic activity [20]. However, Le et al. (2019) reported that *Lactiplantibacillus plantarum* MJM60383, *Lactococcus lactis* MJM60392, *Limosilactobacillus fermentum* MJM60393, and *Lacticaseibacillus paracasei* MJM60396 strains isolated from fermented foods did not show mucinolytic activity [170]. Coimbra-Gomes et al. (2022) also suggested that all strains of acidophilic LAB isolated from fermented Cobrançosa table olives were safe for human consumption, with negative results for mucin degradation, hemolytic activity, and DNase activity [85]. In another study, none of the strains of 59 *enterococci* and 40 non-enterococci showed mucinolytic activity [161]. Considering the health risks, in vivo animal model experiments can be planned in the future to determine the mucinolytic activities of acidophilic LAB with probiotic potential.

## 5. Conclusions

Microorganisms are a group of living creatures that adapt or develop in very different environmental conditions. Acidophilic LAB (such as *Lactobacillus* and *Oenococcus*, which show more acidophilic behavior), which are among the low-pH microorganisms that are attributed as microorganisms that adapt to living in an acidic pH, are the most frequently used microorganisms for food fermentation and preservation. At present, LAB are often preferred as starter cultures due to their unique metabolic properties in the fermentation process under controlled conditions with carefully selected strains. However, it should not be ignored that some acidophilic microorganisms may cause food spoilage and strains with proven safety in the food industry should be preferred. In addition, it is predicted that the use of acidophilic LAB in the natural disposal of wastes generated as a result of industrial applications will become increasingly widespread.

The search for novel, safe, efficient, and promising acidophilic LAB with beneficial effects on human health and the food industry is gaining momentum around the world. Considering the health benefits and antimicrobial and bactericidal activities in foods of acidophilic LAB, which produce compounds that have been shown to have beneficial effects, such as organic acids, B group vitamins, GABA, some enzymes, and bacteriocins, their use is likely to become widespread in the coming years. However, it should not be ignored that some LAB with a low pH can produce BAs, a harmful compound. Antibiotic-resistance gene transfer should also be taken into account when selecting isolates, as there is a risk of the horizontal transfer of these genes through acidophilic LAB. Moreover, the presence of hemolytic, DNase, gelatinase, mucinolytic activities, and virulence genes of these acidophilic microorganisms should be evaluated before they are used in the health and food industry, and it should be demonstrated that their use is safe. For all these reasons, more inclusive studies involving in vitro and in vivo analyses, animal and human subjects should be conducted to investigate the health benefits and safety of acidophilic LAB and the compounds they release. Consequently, this review may direct attention toward the unknown aspects of acidophilic LAB, clarify crucial unanswered questions, and ultimately lead to the development of novel alternative therapies/functional products based on acidophilic probiotic LAB and their metabolites.

## Figures and Tables

**Table 1:** Important species of acidophilic and aciduric LAB with the food sources (adapted from the references [31,40]).

Microorganism	Food in Which İt İs İsolated/Used
*Lactobacillus delbrueckii* subsp. *bulgaricus*	Dairy products
*Ligilactobacillus acidipiscis*	
*Lactobacillus acidophilus*	
*Lactobacillus delbrueckii* subsp. *lactis*	
*Levilactobacillus brevis*	
*Lacticaseibacillus casei*	
*Lactiplantibacillus plantarum*	
*Loigolactobacillus coryniformis* subsp. *coryniformis*	
*Lactobacillus helveticus*	
*Levilactobacillus parabrevis*	
*Lacticaseibacillus paracasei* subsp. *paracasei*	
*Lactiplantibacillus pentosus*	
*Lactiplantibacillus plantarum* subsp. *plantarum*	
*Weissella hellenica*	
*Weisella paramesenteroides*	
*Weisella confusa*	
*Lactobacillus helveticus*	Probiotic Dairy Products
*Lacticaseibacillus casei*	
*Lacticaseibacillus rhamnosus*	
*Limosilactobacillus reuteri*	
*Lactiplantibacillus plantarum*	Sauerkraut
*Lactiplantibacillus plantarum*	Meat, Meat Products
*Companilactobacillus alimentarius*	
*Latilactobacillus curvatus* subps. *curvatus*	
*Lapidilactobacillus dextrinicus*	
*Lacticaseibacillus paracasei* subsp. *paracasei*	
*Lactiplantibacillus plantarum* subsp. * plantarum*	
*Latilactobacillus sakei* subsp. *sakei*	
*Weisella hellenica*	
*Weisella viridescens*	
*Weisella paramesenteroides*	
*Weisella confusa*	
*Lactobacillus acetotolerans*	Vegetables
*Levilactobacillus brevis*	
*Lactobacillus acidophilus*	
*Schleiferilactobacillus harbinensis*	
*Lactobacillus helveticus*	
*Companilactobacillus kimchii*	
*Lentilactobacillus kisonensis*	
*Levilactobacillus parabrevis*	
*Lactiplantibacillus plantarum* subsp. *plantarum*	
*Weisella paramesenteroides*	
*Weisella confusa*	
*Levilactobacillus acidifarinae*	Sourdough
*Lactobacillus amylolyticus*	
*Lactobacillus amylovorus*	
*Lentilactobacillus parabuchneri*	
*Lactobacillus crispatus*	
*Limosilactobacillus fermentum*	
*Companilactobacillus crustorum*	
*Lactobacillus gasseri*	
*Levilactobacillus hammesii*	
*Fructilactobacillus fructivorans*	
*Lactobacillus jensenii*	
*Lactobacillus johnsonii*	
*Lacticaseibacillus manihotivorans*	
*Companilactobacillus mindensis*	
*Limosilactobacillus mucosae*	
*Weisella cibaria*	
*Weisella confusa*	
*Ligilactobacillus acidipiscis*	Fish
*Companilactobacillus alimentarius*	
*Companilactobacillus farciminis*	
*Weisella thailandensis*	
*Lentilactobacillus parabuchneri*	Wine
*Lentilactobacillus hilgardii*	
*Liquorilactobacillus oeni*	
*Lactiplantibacillus pentosus*	
*Liquorilactobacillus cacaonum*	Cocoa
*Lactiplantibacillus fabifermentans*	
*Liquorilactobacillus ghanensis*	
*Liquorilactobacillus nagelii*	
*Secundilactobacillus collinoides*	Fruits
*Liquorilactobacillus mali*	
*Lactiplantibacillus pentosus*	
*Paucilactobacillus suebicus*	
*Agrilactobacillus composti*	Beverages
*Fructilactobacillus fructivorans*	
*Liquorilactobacillus hordei*	
*Latilactobacillus sakei* subsp. *sakeii*	
*Liquorilactobacillus mali*	
*Lentilactobacillus diolivorans*	Cereals
*Limosilactobacillus frumenti*	
*Companilactobacillus farciminis*	Soy

**Table 2:** Overview of the probiotic properties of acidophilic and aciduric LAB in some traditional fermented foods.

Author and Reference	Country	Traditional Fermented Foods	Study Design	Microorganisms	Conclusion
Sadeghi et al. [81] (2022)	Iranian	Dairy products	The probiotic qualities of 144 different strains of LAB were investigated.	-*Lacticaseibacillus paracasei* S23 -*Lactiplantibacillus plantarum* S57 -*Lactiplantibacillus plantarum* S70 -*Lacticaseibacillus casei* S81	These four acidophilic–aciduric LAB show great probiotic potential and antimicrobial activity with their high autoaggregation, coaggregation and hydrophobicity, and high biofilm formation capacity.
Khan et al. [82] (2016)	Korea	Kimchi	The functional properties of *Lactiplantibacillus plantarum* DGK-17 isolated from Kimchi were investigated.	*-Lactiplantibacillus plantarum* DGK-17	This acidophilic microorganism has a strong probiotic potential and antimicrobial activity.
Bin Masalam et al. [6] (2018)	Saudi Arabia	Milk	Thirteen distinct types of raw and fermented milk were used to isolate 93 possible LAB.	*-Lacticaseibacillus casei* MSJ1 -*Lacticaseibacillus casei* Dwan5 -*Lactobacillus plantarum* EyLan2 -*Enterococcus faecium* Gail-BawZir8	In terms of tolerance to acidic pH, bile resistance, and antibacterial activity, it was determined that these four LAB exhibited the best probiotic properties.
Sagdic et al. [83] (2014)	Turkey	Gilaburu	The probiotic potentials of LAB isolated from traditional Turkish fermented European cranberrybush (*Viburnum opulus* L.; Turkish name is gilaburu) fruit juice were determined.	*-Lacticaseibacillus casei* G20a -*Lactiplantibacillus plantarum* G19e	*Lacticaseibacillus casei* (G20a) and *Lactiplantibacillus plantarum* (G19e) were identified as the strains with the highest cell hydrophobicity degrees. *Lactiplantibacillus plantarum* strains were found to be more tolerant to acidic pH than other strains and could grow at pH 2.5. It was concluded that, in addition to the *Lactobacillusplantarum* strain, which is dominant in fermented gilaburu juice, 11 different identified LAB strains can be used as probiotic bacteria.
Margalho et al. [11] (2021)	Brazil	Artisanal cheeses	The probiotic potential of LAB strains (n = 220) isolated from Brazilian artisanal cheeses was investigated.	*-Lactiplantibacillus plantarum* -19 more acidophilic LAB	Twenty acidophilic isolates were evaluated as probiotics because they met these criteria according to their low pH resistance, bile salts, GI tolerance and adhesion properties. Among them, *Lactiplantibacillus plantarum* significantly reduced the number of *Staphylococcus aureus* and *Listeria monocytogenes* pathogens.
Metrouh et al. [84] (2022)	Algeria	Traditional cheeses “Jben”	The probiotic potential of *Lactiplantibacillus plantarum* SJ14 isolated from Algerian traditional cheeses (Jben) was evaluated.	-*Lactiplantibacillus plantarum* SJ14	This low-pH microorganism showed desirable probiotic characteristics.
Coimbra-Gomes et al. [85] (2022)	Portugal	Cobrançosa Table Olives	Nineteen native LAB strains isolated from Cobrançosa table olives were studied.	*-Lactiplantibacillus paraplantarum* i101 -*Lactiplantibacillus pentosus* i53 -*Lactiplantibacillus pentosus* i106	This acidophilic strain showed the highest survival rate.
Liu et al. [86] (2022)	China	Fermented Vegetables	The *Lactobacillus* strains that were obtained from traditionally fermented vegetables were put through an in vitro probiotic test.	*-Lactiplantibacillus plantarum* *-Levilactobacillus brevis* *-Weissella viridescens*	Out of the 74 strains that were isolated from fermented vegetables, 26 demonstrated high survival rates in the presence of bile salts and a low pH. A total of 15 of these strains are *Lactiplantibacillus plantarum* strains, 9 are *Levilactobacillus brevis* strains, and the final two are *Weissella viridescens* strains.
Akmal et al. [87] (2022)	Pakistan	Artisanal Fermented Pickles	Nine *Lactobacillus* strains isolates with promising probiotic potential from fifty different traditional fermented pickle samples were investigated.	*-Lactiplantibacillus plantarum* *-Lacticaseibacillus paracasei* *-Levilactobacillus brevis*	It was determined that three of the strains with probiotic potential belonged to *Lactiplantibacillus plantarum*, five to *Lacticaseibacillus paracasei,* and one to *Levilactobacillus brevis*.
Leite et al. [88] (2015)	Brazil	Kefir grains	In this study, the potential probiotic qualities of 34 LAB were investigated after being isolated from a variety of Brazilian kefir grains.	*-Lacticaseibacillus paracasei* MRS59	This acidophilic strain has been recognized as a promising probiotic candidate.
Lee et al. [66] (2016)	Korea	Kimchi	The probiotic potential of four LAB strains isolated from kimchi was investigated.	-*Lactiplantibacillus plantarum* C182 -Three *Leuconostoc* strains	*Leuconostoc mesenteroides* F27 and *Lactiplantibacillus plantarum* C182 strains can be used as potential probiotics.
Olajugbagbe et al. [89] (2020)	Nigeria	Wara	The probiotic potential of *Pediococcus acidilactici* isolated from Wara, a Nigerian unripened soft cheese from cow milk, was studied.	-*Pediococcus acidilactici*	It was concluded that this strain, which was demonstrated to survive at pH 2 and 1.5% bile salt concentration, and to have a high auto-aggregation ability and hydrophobicity, might be a useful probiotic for the development of functional food products.

## Data Availability

Not applicable.
